# A rhodanine agent active against non-replicating intracellular *Mycobacterium avium *subspecies *paratuberculosis*

**DOI:** 10.1186/1757-4749-1-25

**Published:** 2009-12-23

**Authors:** Tim J Bull, Richard Linedale, Jason Hinds, John Hermon-Taylor

**Affiliations:** 1 Department of Cellular and Molecular Medicine, St George's University, Cranmer Terrace, London, SW17 0RE, UK; 2 Division of Nutritional Sciences, Franklin-Wilkins Building, King's College London, 150 Stamford Street, London SE1 9NH, UK

## Abstract

**Background:**

Antibiotic therapy targeting chronic mycobacterial disease is often ineffective due to problems with the emergence of drug resistance and non-replicating persistent intracellular antibiotic resistant phenotypes. Strategies which include agents able to enhance host cell killing mechanisms could represent an alternative to conventional methods with the potential for host clearance if active against dormant phenotypes. Investigations of agents with potential activity against non-replicating mycobacteria however are restricted due to a need for assays that can assess bacterial viability without having to culture.

**Results:**

This study describes the development and use of a pre16S ribosomal gene RNA/DNA ratio viability assay which is independent of the need for culture, supported by a novel thin layer accelerated mycobacterial colony forming method for determining viability and culturability of MAP in intracellular environments. We describe the use of these tools to demonstrate intracellular killing activity of a novel rhodanine agent (D157070) against the intracellular pathogen *Mycobacterium avium *subspecies *paratuberculosis *(MAP) and show that the culturability of MAP decreases relative to its viability on intracellular entry suggesting the induction of a non-culturable phenotype. We further demonstrate that D157070, although having no direct activity against the culturability of extracellular MAP, can bind to cultured MAP cells and has significant influence on the MAP transcriptome, particularly with respect of δ^L ^associated genes. D157070 is shown to be taken up by bovine and human cells and able to enhance host cell killing, as measured by significant decreases in both culturability and viability of intracellular MAP.

**Conclusions:**

This work suggests that pre16srRNA gene ratios represent a viable method for studying MAP viability. In addition, the rhodanine agent D157070 tested is non-toxic and enhances cell killing activity against both growing and latent MAP phenotypes.

## Background

The pathogenic strategy of bacteria associated with chronic disease often progresses by the inhibition of host innate immune mechanisms that result in activation of host intracellular killing and facilitating long term intracellular persistence [1]. Some of these pathogens also have the ability to convert into a viable non-replicating phenotype on cell entry [2]. These forms are often stable for long periods with altered transcriptomic turnover of cell wall constituents [3,4]. As a consequence, the abundance of bacterial antigens presented to the host is decreased reducing immune recognition, promoting anergy and increasing intracellular longevity. Antibiotic therapy may have limited efficacy during such chronic infective conditions, particularly when the activity of the agent targets essential features of replicating organisms such as cell wall bio-genesis [5,6]. Chemotherapy to achieve clearance of these chronic infections often requires prolonged treatment regimes with the associated problems of toxicity, patient compliance and the emergence of drug resistance.

*Mycobacterium avium *subspecies *paratuberculosis *(MAP) is a proven gut pathogen that is the cause of chronic enteritis (Johne's disease JD) in many animals including sub-human primates [7]. It can be detected and cultured from both blood and gut tissue of up to 40% of normal humans [8] and in some studies has be found in over 80% of patients with Crohn's Disease (CD) [9]. MAP has the capacity to dysregulate host immune systems, a critical factor in the development of CD in genetically susceptible patients [10]. In some cases of CD anti-MAP therapy using antibiotics with enhanced activity against these organisms has resulted in clinical remission with healing of the inflamed gut and apparent MAP clearance [5,11]. However only a proportion of CD patients respond and the approach is open to all the problems of bacterial latency and the development of microbial drug resistance seen in the treatment of chronic fibrotic lung disease due to closely related organisms such as *Mycobacterium avium *subspecies *avium *[12,13]. Such chronic clinical infections require novel chemotherapeutic approaches particularly those in which the agent is active against non-replicating phenotypes.

One of the mechanisms used by pathogenic mycobacteria to inhibit intracellular killing by the host and provide a stable environment for persistence, is through the action of microbial alkylhydroperoxidase reductase subunit C (AhpC) [14]. In most acidic conditions, this is a major secreted protein and is concentrated in phagosomes where it is active against reactive nitrogen intermediates (RNI) derived by the host from nitric oxide [15]. Activity of the AhpC gene product, which in MAP is encoded by MAP1589c, requires it to be in a reduced form [16]. This is achieved through a pathogen-derived renewal system involving an oxidation/reduction cascade with three other genes called *ahp*D (MAP1588c), *dla*T (MAP1956) and *lpd*A (MAP3424). Previous work has shown that this cascade can be irreversibly inhibited by the binding of a group of rhodanine derivatives to the DlaT component found in the mycobacterial cell wall [17]. These chemotherapeutic agents have *in vivo *activity against wild type *Mycobacterium tuberculosis *(MTB) but are inactive against DlaT knockout MTB mutants and are thought to act by enhancing the normal killing capacity of the host

In this work we have investigated the activity of one of these rhodanine agents (D157070) in enhancing the intracellular killing of MAP. D157070 is a propanolic ester derivative of an active rhodanine component (3-((Z)-5-((5-(2-chlorophenyl)furan-2-yl)methylene)-4-oxo-2-thioxothiazolidin-3-yl)benzoic acid) modified to potentiate phagosomal cell entry [17]. To measure the effect of D157070 on intracellular mycobacterial viability we have developed an assay which is independent of the need for culture supported by a novel thin layer accelerated MAP colony forming culture method. Using these assays we show that the culturability of intracellular MAP is much lower than its viability, suggesting the induction of a non-culturable phenotype on cell entry. We also show that D157070 treatment of MAP in vitro has significant influence on the MAP transcriptome but does not reduce viability. In contrast D157070 treatment of intracellular MAP causes decreases in both culturability and overall viability suggesting that D157070 is active within cells against both actively growing and latent MAP phenotypes.

## Results

### D157070 has no antibacterial activity in vitro against MAP as measured by MAP culturability and viability assays

**Figure 1:**
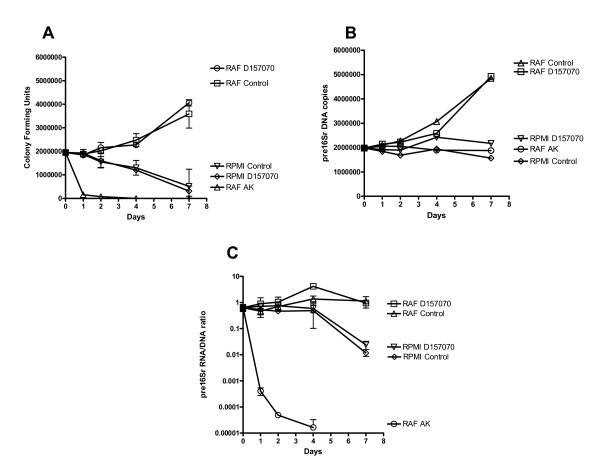
**Pre16Sr DNA copy number, Culture (cfu), and Viability curves of MAP K10 in media**. MAP K10 growth curves (average of three experiments) in RPMI medium with and without D157070 treatment and in RAF medium, with or without D157070 treatment or Amikacin treatment plotted as A) copies of pre16Sr DNA B) colony forming units C) pre16Sr RNA/DNA ratio.

Comparing culture samples of total cfu with estimations derived from pre16Sr DNA qPCR at Day 0 (Figure 1A &1B), the thin layer method showed growth was possible in more than 80% of organisms present in an exponentially growing liquid culture inoculum. Results were available more rapidly than conventional methods with micro-colonies visible at 14 days (samples read at four weeks, showed no significant increase in cfu). An estimation of the proportion of large and small colonies was made in each sample which showed the degree of clumping remained consistent throughout the experiment at around 5% (data not shown).

The pre16Sr RNA/DNA ratio was found to be approximately 1:1 in exponential growth culture and did not appear to fluctuate significantly as growth occurred suggesting that expression was constant during this growth phase. As expected, treatment of cultures with an antibiotic (Amikacin) immediately caused a significant decrease in both cfu and ribosomal turnover with both becoming effectively negative after 4 days treatment (Figure 1B &1C). This suggested that viability and culturability were being effectively assayed. Introduction of MAP into media that is not supportive of MAP growth (RPMI), after an initial lag phase, resulted in a decrease in ribosomal turnover (Figure 1C) and proportionately a significantly larger decrease in culturability (Figure 1B) suggesting that these assays were not measuring identical phenomena.

Culture of MAP-K10 in control RAF medium showed a steady increase in growth as expected. Proportionate increases in cfu and pre16Sr DNA copy number were consistent with a doubling of the initial inoculum over the 7 day experiment. Ribosomal turnover ratio however remained relatively constant throughout. MAP growth was not observed in the RPMI medium with the pre16Sr DNA copy number remaining constant. There was a steady drop in culturability after 1 day but a significant decrease in ribosomal turnover (RNA/DNA pre16Sr ratio) did not occur until 7 days (Figure 1C), suggesting that culturability was lost prior to viability. Treatment with D157070 showed no significant differences in either culturability or ribosomal turnover in both RAF and RPMI media suggesting that this agent is not directly active towards the viability of MAP in vitro. All D157070 treated MAP cultures retained the orange pigmentation associated with the agent, suggesting cell wall binding of the drug had occurred.

### D157070 treatment in vitro induces transient increases in expression of MAP genes associated with δL but not the ahpC oxidation/reduction cascade

MAPAC microarray analysis of in vitro MAP transcriptome profiles from RAF culture treated for up to 3 days with D157070 and compared with untreated controls identified 63 differentially regulated MAP genes with a significant (p < 0.05) and greater than two-fold change in expression between Day 0 and Day 1-3. Thirty six of these genes were located in sequentially adjacent clusters within the genome, representing 8 up-regulated and 7 down-regulated putative operons (Table 1). Of the 8 up-regulated operons, homologues from genes in 3 operons are already known to be regulated in other organisms by the δ^L ^transcription factor (MAP1369-MAP1371; MAP2642-MAP2644; MAP2940c-2942c) [18,19]. Three further up-regulated operons were located immediately upstream to the δ^L ^gene (MAP4202-MAP4205; MAP4206c-MAP4207c; MAP4208-MAP4211) including the δ^L ^related factor RslA (MAP4202) suggesting that δ^L ^related control is an important factor in MAP in vitro response to D157070. Of particular interest was also the down- regulation of a set of mammalian cell entry genes (*mce1*) associated with virulence (MAP3606-MAP3607) as well as the MAP *katG *gene (MAP1668c) which is an essential factor involved in intracellular MAP persistence. There were no significant differential changes in expression levels observed for *ahpC *(MAP1589c) or *ahpD *(MAP1588c) although these were constitutively expressed. Other genes in the AhpC oxidation/reduction cascade including *lpdA *(MAP3424) and the MAP homologue *dlaT *(MAP1956) of the proposed substrate for D157070 in MTB, were similarly unaffected.

**Table 1:** Differentially regulated MAP genes after D157070 treatment

		Fold Change			
				
Gene Name	p-value	Day 1	Day 3	Putative function	MTB homologue
**Upregulated**					

MAP0493c (acrR)	0.044	2.92	1.42	transcriptional regulator	NS
MAP0494	0.011	3.37	1.66	hypothetical protein	NS
MAP0495c	0.002	2.24	1.26	hypothetical protein	Rv3572
MAP0496c	0.016	3.10	1.69	oxidoreductase	Rv3571

MAP1369 (pks10)	0.044	2.43	1.77	polyketide synthase	Rv1660
MAP1371 (pks8)	0.009	2.47	1.93	polyketide synthase	Rv1662

MAP1724c (yceJ)	0.011	4.21	1.94	cytochrome like protein	NS
MAP1725c (srpA)	0.021	5.02	2.81	catalase like protein	NS

MAP2634c (smtA)	0.044	2.78	2.67	hypothetical protein	Rv1147
MAP2635c (mmpl13)	0.002	3.73	1.93	mmpL protein	Rv1146/5

MAP2642	0.001	9.00	3.59	naringenin-chalcone synthase	Rv1660
MAP2643	0.001	7.93	3.20	methyltransferase	Rv1139c
MAP2644	0.001	4.90	2.23	oxidoreductase	Rv1138c

MAP2940c (dxr)	0.016	2.49	2.43	reductoisomerase	Rv2870c
MAP2941c	0.009	6.75	3.78	cytochrome C biogenesis protein	Rv2877c
MAP2942c (mpt53)	0.031	7.43	3.07	Mpt53	Rv2878c

MAP3545	0.016	2.45	1.73	oxidoreductase	Rv3230c
MAP3546 (desA3_2)	0.016	3.02	1.90	DesA3_2	Rv3229c

MAP4201 (δ^L^)	0.018	2.60	1.53	RNA polymerase sigma-L factor	Rv0735
MAP4202 (rslA)	0.019	2.50	1.59	hypothetical protein	Rv0736
MAP4203	0.007	3.34	2.19	oxidoreductase	NS
MAP4204	0.012	2.74	2.01	hypothetical protein	Rv1888c
MAP4205 (UbiE)	0.002	3.94	2.25	methyltransferase	NS
MAP4206c (lolC)	0.017	4.06	3.36	efflux ABC transporter	NS
MAP4207c (lolD)	0.003	5.91	2.77	ABC-type transporter	NS
MAP4208 (ilvB)	0.002	6.93	3.40	acetolactate synthase	Rv3740c
MAP4209	0.000	7.41	3.97	transcriptional regulator	NS
MAP4210 (fabH)	0.005	7.04	4.24	3-oxoacyl-ACP synthase III	Rv0533c
MAP4211 (pat)	0.003	6.19	4.82	histidinol-phosphate aminotransferase	Rv3772

**Downregulated**					

MAP1553c (fadE14)	0.009	2.27	1.88	putative acyl-CoA dehydrogenase	Rv1346
MAP1554c (fadD33_2)	0.018	2.50	2.50	acyl-CoA synthase	Rv1345
MAP1555c	0.017	2.50	1.45	acyl carrier protein	Rv1344

MAP1668c (katG)	0.016	2.50	1.81	catalase/peroxidase	Rv1908c

MAP1996 (fabD)	0.022	1.92	0.76	malonyl CoA-acyl carrier protein	Rv2243

MAP2331c (acpS)	0.016	2.32	2.85	holo- [acyl-carrier-protein] synthase	Rv2523c
MAP2332c (fas)	0.002	2.70	4.76	fatty acid synthase	Rv2524c

MAP3177	0.018	2.70	2.22	hypothetical protein	Rv3129
MAP3178c	0.016	3.12	1.12	hypothetical protein	NS
MAP3179c	0.044	3.33	1.61	hypothetical protein	Rv3134c

MAP3272	0.027	2.70	1.22	pyridoxamine 5'-phosphate oxidase	Rv3129
MAP3273c	0.027	3.22	1.63	hypothetical protein	Rv3131

MAP3600	0.032	1.85	2.04	hypothetical protein	Rv0166
MAP3601 (fadD5)	0.006	2.43	4.00	acyl-CoA synthase	Rv0166
MAP3602	0.047	2.00	2.04	hypothetical protein	Rv0167

MAP3606	0.016	2.38	3.44	mce-family protein mce1c	Rv0171
MAP3607	0.017	1.96	1.63	mce-family protein mce1d	Rv0172

MAP3671	0.009	2.70	1.96	transcriptional regulator	Rv0232

Selected gene list with putative function predictions showing fold change of upregulated and downregulated operons (p < 0.05) of Day 1 D157070 treatment normalized to untreated control. Genes with homologues in MTB are indicated (NS = no significant homology).

### D157070 treatment of MAP inoculated into activated and non activated macrophage cell cultures induces enhanced host killing as measured by MAP culturability and viability assays

**Figure 2:**
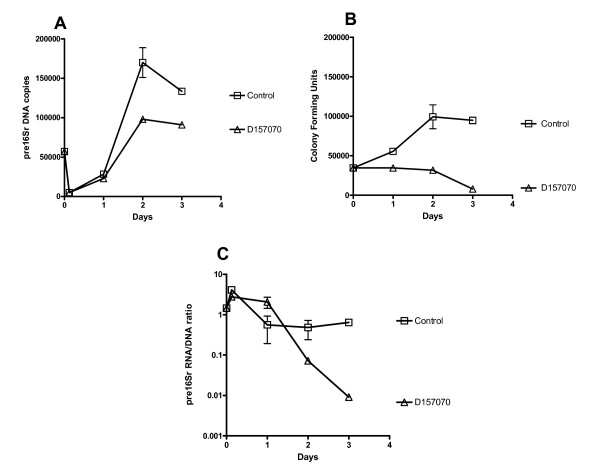
**Pre16Sr DNA copy number, Culture (cfu), and Viability curves of MAP K10 in BOMAC**. MAP K10 growth curves (average of three experiments) infected into BOMAC cell line at MOI 1:1 with or without D157070 treatment plotted as A) copies of pre16Sr DNA B) colony forming units C) pre16Sr RNA/DNA ratio.

BOMAC cell lines were fully permissive for MAP infection and after an initial period of uptake, rapid growth of MAP occurred which peaked at 2 days (Figure 2A &2B). MAP DNA increased in proportion to cfu whilst ribosomal turnover increased transiently on cell entry but was only minimally elevated overall. MAP infected BOMACS treated with D157070 showed no change in cfu by Day 2 and an 80% reduction by Day 3. Pre16Sr DNA copy number increased until Day 2 but were significantly reduced compared with untreated controls (p = <0.001). Ribosomal turnover fell sharply after Day 1 consistent with loss of viability and continued to fall throughout the length of the experiment (Figure 2C).

**Figure 3:**
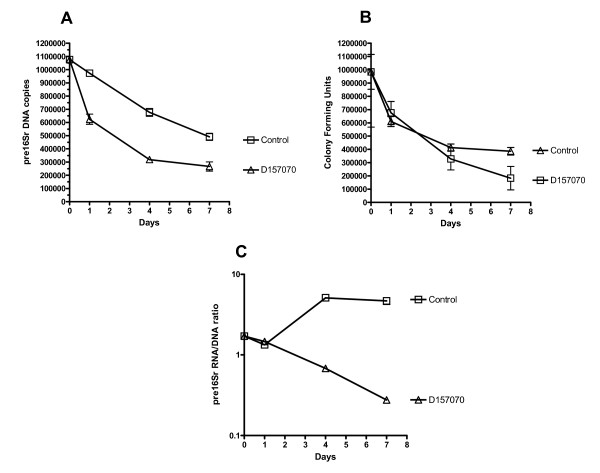
**Pre16Sr DNA copy number, Culture (cfu), and Viability curves of MAP K10 in THP1**. MAP K10 growth curves (average of three experiments) infected into THP1 cell line at MOI 1:1 with or without D157070 treatment plotted as A) copies of pre16Sr DNA B) colony forming units C) pre16Sr RNA/DNA ratio.

THP1 cell lines were pre-activated before MAP infection and were therefore primed for MAP killing which was evident after infection. Only 30% of the initial inoculum remained culturable at 4 days, after which the reduction in culturability slowed (Figure 3A and 3B). Pre16Sr DNA copy number also fell but at a significantly slower rate than cfu resulting in 30-50% higher values relative to cfu. The ribosomal turnover ratio per MAP cell increased initially on cell entry, peaking at 4 days (Figure 3C) suggesting a differential MAP transcriptomic response to the change in environment when compared with MAP infection into BOMAC bovine cells (Figure 2C). In striking contrast, pre16Sr DNA copy number, cfu and ribosomal turnover ratios of infected THP1 cells treated with D157070, decreased steadily and significantly faster than untreated controls, resulting in a minimal ribosomal turnover with only 20% of the original inoculum being viable or culturable by Day 7.

## Discussion

The ability of slow growing mycobacterial pathogens to adopt a non-culturable but viable phenotype is consistent with their capacity for chronic intracellular existence. This represents an important attribute of their pathogenicity, providing them with a means for long term persistence and ability to influence on the host cell whilst escaping clearance by therapies that act on microbial cell division. It is clear that MAP belongs to this group. It can shut down cell division, transforming from an already very slow growing organism into one in a state of latency [20]. This is particularly evident in human MAP infection where changes in the cell wall, low microbial loads and unculturable phenotypes predominate [2,3]. These phenotypes are associated with major alterations in transcriptional activity although as yet the triggers for them are not known. In MTB they are associated with the response to hypoxia [21,22].

Studies using rapidly growing pathogens would normally regard demonstrable growth in the laboratory as the essential indicator of viability. The existence of non-culturable phenotypes of MAP create some unique experimental difficulties requiring novel assays to distinguish between the capacity for growth *in vitro *(defined here as culturability) and a measure of viability that is independent of the need for culture. In this study we have addressed this problem with the development of an optimised thin layer culture method to determine culturability and a separate MAP-specific RNA/DNA pre16Sr copy ratio assay as a measure of MAP ribosomal turnover and viability. Both methods were validated using a conventional antibiotic *in vitro *killing assay and showed similar rapid responses to loss of viability. Multiple copies of the ribosomal operon in other bacteria such as *E.coli *[23] ensure that in nutrient rich media the ribosomal turnover can adapt to support increases in growth rate. The MAP genome encodes only a single ribosomal operon and like all slow growing mycobacterial species contains only one promoter to drive its transcription [24]. We show that MAP inoculated into media normally unsupportive of growth demonstrate a decrease in ribosomal turnover which is preceded by a proportionally larger decrease in culturability, suggesting that starvation induces a loss in culturability before viability. In conventional culture media we confirm the findings of others [25] showing that ribosomal turnover rates are not related to mycobacterial growth phase and further show that in MAP this rate is relatively stable. One possible indication from this is that the basal ribosomal turnover in MAP is set at a level sufficient for essential protein synthesis in the ground state but that there is a lag in elevation of turnover in response to an increased demand from divisional activity which thus acts as a limiting factor on growth. Decreases below the basal turnover ratio (as seen in these studies after prolonged starvation or antibiotic treatment) would thus represent a rate of transcriptional activity insufficient to retain viability.

In accordance with previous observations [26,27] we found that a bovine derived macrophage cell line was fully permissive to MAP. Cell entry induced a transiently higher rate of MAP division (increases in cfu and DNA levels) than that observed in parallel conventional cultures. Significantly the rate of ribosomal turnover varied only minimally during the whole course of the infection confirming that the BOMAC cell line was unable to kill MAP over the time interval of the study. In striking contrast, ribosomal turnover in MAP increased sharply after initial cell entry into human macrophages (THP1). In these activated cells MAP killing was expected and indeed as in RPMI culture, both culturability and the total amount of MAP DNA decreased at significantly different rates. This indicated that despite being activated, THP1 cells were only able to kill up to 50% of MAP cells after cell entry with an increasing proportion of these (20%) becoming viable non-culturable. These results suggest that MAP phenotypic responses to intracellular environments are dependant on the degree of hostility encountered. Increasing transcriptomic activity in hostile environments may be envisaged as reflecting the deployment of MAP derived survival mechanisms to de-activate host cell killing leading to the inhibition of MAP division but may also signal the emergence of the non-culturable phenotype.

The introduction of D157070 into the three test systems (BOMAC, THP1 and *in vitro *Culture) gave a varied response. In extracellular *in vitro *conditions there was no observable effect on either culturability or ribosomal turnover suggesting that this agent does not act directly on MAP viability. The lack of bacteriostatic or bacteriocidal activity is consistent with previous studies showing that dlaT gene functionality is not essential for mycobacterial viability or growth in nutrient rich conventional media [17]. MAP growing exponentially in culture medium showed differential transitional transcriptional profiles on treatment with D157070 that were sustained for up to 3 days. This included 63 genes clustered in 8 up-regulated operons and 7 down-regulated operons. Previous studies have suggested that D157070 binds with the dihydrolipoamide transferase DlaT, probably on the surface of the mycobacterial cell and that this leads to alterations in AhpC functionality through a cascade involving the genes AhpD and LpdA [16]. Binding to MAP cells was indeed evident because treated MAP cultures retained the pigment associated with the agent. However none of the differentially transcribed genes observed in this study could be related to AhpC activation. Putative functions of the genes observed to be upregulated were mostly those associated with lipid metabolism and membrane transport mechanisms. Some were also linked to operons known to under the control of the global transcription regulator δ^L ^which is an important regulator of cold stress in other organisms [28,29]. Interestingly, δ^L ^regulated genes have been related to virulence in MTB [18,19,30] and intracellular infection in MAP [31]. Genes that were down regulated included the mce1 operon, shown to be involved in mycobacterial growth control [32] and *kat*G associated with oxidative stress and intracellular persistence [33]. The means by which these regulatory changes occur are unknown but they could be the result of alternative mechanisms of action of D157070 other than through AhpC. The action of D157070 on δ^L ^controlled lipid and cell wall synthesis in MAP could alter host cell recognition. The reduction in expression of several genes associated with virulence and persistence in mammalian cells may also represent a direct influence of D157070 on the capacity of MAP for intracellular survival.

Significantly D157070 was active against MAP in both BOMAC and THP1 cell lines. Both culturability and ribosomal turnover decreased significantly compared with controls. In BOMAC cells, whilst total MAP pre16Sr DNA increased up to Day 2, the culturability did not and MAP ribosomal turnover began to drop significantly after Day 1. This delay probably reflects the initial unactivated state of the cell line and represents the need of the cell to initiate RNI related killing processes. In THP1 cells, killing was already evident at Day 1 and both culturability and total MAP pre16Sr DNA fell proportionally. However the clearance of MAP from cells in response to D157070 as measured by the total MAP pre16Sr DNA was significantly greater than by THP1 cells alone. In addition, the ribosomal turnover showed a steady and significant decrease throughout the time course of the experiment. This is indicative of enhanced killing by the activated cells and probably indicates that D157070 was able to prevent MAP from blocking intracellular killing processes. The parallel decrease in both total DNA and cfu observed in these experiments also suggests that MAP invasion into cells treated with D157070 did not convert to the unculturable phenotype.

## Conclusions

We have developed culturability and viability assays which demonstrate that D157070 initiated enhanced host directed killing of intracellular MAP by host cells. D157070 was well tolerated by host cells and its activity was independent of the need for intracellular MAP growth or the pre-activation of host cell killing mechanisms. D157070 was readily associated with cultured MAP cells but showed no direct anti-MAP activity in conventional extracellular culture. In culture, MAP exhibited transcriptional responses to exposure to D157070 but these were not associated with genes linked to the MAP AhpC oxidation/reduction cascade. This is not in conflict with the previously proposed mechanism of action but suggests that D157070 may have alternative or complementary mechanisms of action in the intracellular environment other than through AhpC inactivation

This rhodanine agent represents a novel approach to anti-MAP chemotherapy that is active against both dividing and dormant intracellular infection. Further studies in animals are now required to investigate possible toxicity issues and the efficacy of clearance in chronic models of infection. This type of approach offers a promising potential as therapy for chronic low load MAP infections in humans.

## Methods

### Accelerated MAP culture by microaerophilic thin layer colony counting

The thin layer method was developed to avoid using highly oxygenated conventional solid media that previously had produced variable colony counts from liquid media possibly influenced by clumping. The new method was less likely to dry out than conventional plates and provided a microaerophilic environment enveloping the bacteria with growth media which allowed discrete colony formation that facilitated counting of micro-colonies by microscopy. Culture media consisted of 9 mls of RAF base medium (d-L asparagine 5 g/l, potassium dihydrogen phosphate 2 g/l, magnesium sulphate. 7H_2_O 1 g/l, tri-ammonium citrate 2 g/l, sodium chloride 2 g/l, D-glucose 10 g/l, ammonium iron (III) citrate brown 75 mg/l, glycerol 0.5% (w/v), Mycobactin J 2 mg/l: adjusted to pH 5.7) supplemented with 1.5% agar noble, 10% foetal bovine serum (inactivated), 25 μg/ml Amphotericin B, 100 μg/ml Naladixic acid, 100 μg/ml Vancomycin set in 25 cm^2 ^tissue culture flasks (Nunc, UK) with non filtered caps. Samples of MAP preparations were serially diluted to a final volume of 1 ml RAF base medium then rapidly mixed with 1 ml of warm RAF base medium plus 1.5% agar maintained in a 45°C water bath and pipetted over the set RAF basal layer. Caps were made air tight and cultures incubated at 37°C. Colony counts were made at 14 days and 28 days using an inverted microscope (200 × magnification). Preparation of MAP samples from culture were made by centrifuging at 4,000 ×g for 10 mins. Cell lines containing MAP were initially treated for 1.5 hrs with Amikacin (200 μg/ml) to kill extracellular MAP, washed briefly in PBS then, separated by differential cell lysis for 1 minute in 1% SDS in 50 mM Hepes: 0.05% Nonidet P40 buffer, 1 U Benzonase (Cat:70746-3, VWR, UK) followed by centrifugation as above.

### Quantitation and viability assays using pre16Sr DNA and pre16Sr RNA

Viability was determined using a RNA/DNA copy ratio of a MAP genomic region immediately preceding the single copy ribosomal operon, referred to here as pre16Sr. This genomic region was chosen because maintenance of ribosomal renewal is essential in both latent and growing cells [34]. In addition, the pre16Sr region is cleaved during ribosome assembly expression and is thus constitutively expressed but transiently retained [25,35]. DNA and RNA were extracted from equal aliquots of pelleted MAP sample preparations as described above. For DNA extraction, MAP were resuspended in 600 μl MLB buffer (440 mM NaCl, 1% SDS, 10 mM TrisHCl, 1 mM EDTA pH 8.0), lysed by mechanical disruption at 6.5 ms^-1 ^in a bead B matrix (Qbiogene, UK) for 50 secs, briefly iced, extracted with a standard Phenol/chloroform protocol, washed in 70% ethanol, precipitated with sodium acetate and 100% ethanol and resuspended in RNAse/DNAse free water. MAP samples for RNA extraction were resuspended in 600 μl MELB buffer (1% 2-mercaptoethanol, 440 mM NaCl, 1% SDS, 10 mM TrisHCl, 1 mM EDTA pH 8.0), then lysed by mechanical disruption at 6.5 ms^-1 ^in a bead B matrix (Qbiogene, UK) for 50 secs, briefly iced, extracted with Trizol/chloroform, precipitated with 80% volume isopropanol at -80°C for 1 hour, washed in 70% ethanol then resuspended in RNAse/DNAse with 2 U DNAse I (Invitrogen, UK) for 30 mins at 37°C. This was followed by a second Trizol/chloroform extraction, isopropanol precipitation at -80°C for 1 hour, washing in 70% ethanol and resuspension in DNAse/RNAse free water. cDNA preparations for qPCR reactions were generated using a Superscript II polymerase kit (Invitrogen, UK) according to the manufacturers instructions. All qPCR's consisted 12.5 μl Power SYBR green mastermix (Applied Biosystems, Cat 4368706), 1 μl primer mix (2 pMoles pre16SrRNA.R GCGCAGCGAGGTGAATTT, 2 pMoles pre16SrRNA.F TTTGGCCATACCTAGCACTCC), 9.5 μl H_2_O and 2 μl DNA or cDNA sample per reaction mix, cycling at 1 cycle at 95°C:15 mins; 40 cycles at 95°C:30 secs, 58°C: 1 min, 72°C:1 min with data collection at 76°C (10 secs) in a Mx3000P qPCR cycler (Stratagene, UK). Sample copy numbers were estimated by using a dilution curve of a control stock total genomic DNA MAP K-10 preparation serial diluted to contain between 1 × 10^6 ^and 100 pre16Sr RNA copies.

### MAPAC Microarray

Parallel inoculums of MAP-K10 were made into RAF medium plus 5 μM D157070 and sampled at 1 and 3 days. Three independent replicate experiments performed and the control untreated cultures received DMSO only (solvent for D157070). Total MAP RNA was differentially extracted as described above, cDNA generated, labelled and hybridized as previously described onto individual MAPAC arrays for each sample along with a stock MAP K10 genomic DNA control labelled with a separate dye to normalize signal strengths in a common reference design. MAPAC microarray analysis was performed to derive MAP transcriptomic profiles of each time point. Statistical analysis of the gene expression profiles at each time point identified differentially expressed genes that were up or down-regulated significantly greater than 2 fold at day 1 after treatment relative to a normalized untreated control data set.

Briefly, 1.5 μg of DNA control was labelled by random priming with Klenow polymerase to incorporate Cy3 dCTP and 3 μg of cDNA sample was labeled by random priming with SuperScript reverse transcriptase with Cy5 dCTP (GE Healthcare) using standard protocols [36]. Cy3 and Cy5 labelled samples were co-purified through a Qiagen MinElute column (Qiagen), mixed with a formamide-based hybridization solution (1× MES, 1 M NaCl, 20% formamide, 0.02 M EDTA, 1% Triton) and denatured at 95°C for 2 min. The labeled sample was loaded on to a prehybridised (3.5× SSC, 0.1% SDS, 10 mg/ml BSA) microarray under two 22 × 22 mm LifterSlips (Erie Scientific, UK), sealed in a humidified hybridization cassette (Corning) and hybridised overnight by immersion in a water bath at 55°C for 16-20 h. Slides were washed once in 400 ml 1× SSC 0.06% SDS at 55°C for 2 min and twice in 400 ml 0.06× SSC for 2 min. Microarrays were scanned using an Affymetrix 428 scanner, and signal intensity data were extracted using BlueFuse for Microarrays v3.5 (BlueGnome, Cambridge UK). The intensity data was further post-processed using BlueFuse to exclude both controls and low confidence data (p < 0.1) prior to normalisation using global median centering. Further analysis of the normalised data was undertaken using GeneSpring 7.3.1 (Agilent Technologies). Averaged normalised data from each of the 3 separate biological replicates were further analysed to select genes with significant changes in gene expression in response to D157070 treatment. One-way ANOVA, with a p-value cut-off of 0.05 and including Benjamini and Hochberg False discovery rate correction, was applied to those genes showing a greater than 2-fold change in expression. This identified genes with a significant increase or decrease at Day 1 or Day 3 after treatment compared to untreated controls. Fully annotated microarray data have been deposited in BμG@Sbase (accession number E-BUGS-92; http://bugs.sgul.ac.uk/E-BUGS-92) and also ArrayExpress (accession number E-BUGS-92).

### Activity of D157070 on extracellular MAP cultures

MAP strain K-10 (ATCC BAA-968) was grown into exponential growth phase (approximately 4 weeks) in RAF liquid culture then inoculated into either liquid RAF medium or a tissue culture medium unable to support MAP growth (RPMI). Cultures were treated at time 0 with either an antibiotic (Amikacin), D157070 dissolved in DMSO, or DMSO alone as a control. Samples were taken in triplicate at 0, 1, 2, 4 and 7 days post treatment and investigated for the number of mycobacterial cells (cfu) and their viability using the method described above.

### Activity of D157070 on intracellular MAP

MAP K-10 was infected into non-activated bovine macrophage (BOMAC) and activated human macrophage cell lines (THP1) at an MOI of 1:1. Experiments were performed in triplicate and samples were assayed for cfu and viability from day 0, 1, 4 and 7 in THP1 cells and Day 0, 1, 2 and 3 in BOMAC cells. Test infected cells were treated with 5 μM D157070 which was replaced every 48 hrs in fresh culture medium. Parallel sets of non-infected cells treated with D157070 showed no toxicity over the test period.

## Competing interests

The authors declare that they have no competing interests.

## Authors' contributions

Experiments were designed and performed by TJB and RL. Microarray data analysis was performed by JH. Study conception and writing of the manuscript was by TJB and J-HT.
